# Assessment of the diagnostic and prognostic relevance of ACAT1 and CE levels in plasma, peritoneal fluid and tumor tissue of epithelial ovarian cancer patients - a pilot study

**DOI:** 10.1186/s12885-022-09476-6

**Published:** 2022-04-10

**Authors:** Vijayalakshmi Ayyagari, Maio Li, Zvi Pasman, Xinjia Wang, Somaja Louis, Paula Diaz-Sylvester, Kathleen Groesch, Teresa Wilson, Laurent Brard

**Affiliations:** 1 Department of Obstetrics and Gynecology, Southern Illinois University School of Medicine, Springfield, Illinois USA; 2 Department of Chemistry, Illinois College, Jacksonville, Illinois USA; 3 Center for Clinical Research (CCR), Southern Illinois University School of Medicine, Springfield, Illinois USA; 4 Simmons Cancer Institute, Southern Illinois University School of Medicine, Springfield, Illinois USA

**Keywords:** Epithelial ovarian cancer, ACAT1, CE, Ki67

## Abstract

**Background:**

Abnormal accumulation of acyl-CoA cholesterol acyltransferase-1 (ACAT1) and ACAT1-mediated cholesterol esterified with fatty acids (CE) contribute to cancer progression in various cancers. Our findings of increased CE and ACAT1 levels in epithelial ovarian cancer (EOC) cell lines prompted us to investigate whether such an increase occurs in primary clinical samples obtained from human subjects diagnosed with EOC. We evaluated the diagnostic/prognostic potential of ACAT1 and CE in EOC by: 1) assessing ACAT1 and CE levels in plasma, peritoneal fluid, and ovarian/tumor tissues; 2) assessing diagnostic performance by Receiver Operating Characteristic (ROC) analysis; and 3) comparing expression of ACAT1 and CE with that of tumor proliferation marker, Ki67.

**Methods:**

ACAT1 protein levels in plasma, peritoneal fluid and tissue were measured via enzyme-linked immunosorbent assay. Tissue expression of ACAT1 and Ki67 proteins were confirmed by immunohistochemistry and mRNA transcript levels were evaluated using quantitative real-time polymerase chain reaction (qRT-PCR). CE levels were assessed in plasma, peritoneal fluid (colorimetric assay) and in tissue (thin layer chromatography).

**Results:**

Preoperative levels of ACAT1 and CE on the day of surgery were significantly higher in tissue and peritoneal fluid from EOC patients vs. the non-malignant group, which included subjects with benign tumors and normal ovaries; however, no significant differences were observed in plasma. In tissue and peritoneal fluid, positive correlations were observed between CE and ACAT1 levels, as well as between ACAT1/CE and Ki67.

**Conclusions:**

ACAT1 and CE accumulation may be linked to the aggressive potential of EOC; therefore, these mediators may be useful biomarkers for EOC prognosis and target-specific treatments.

**Supplementary Information:**

The online version contains supplementary material available at 10.1186/s12885-022-09476-6.

## Background

Highly predictive, prognostic biomarkers are essential for developing targeted treatment strategies for epithelial ovarian cancer (EOC). Current approaches for the treatment of EOC are not completely effective as disease recurrence is common. The failure of these therapeutics can be attributed to various escape mechanisms used by metastatic cancer cells [1]. Multiple lipidogenic and cholesterogenic pathways that regulate tumor growth and metastasis are affected in many human cancers including EOC [2]. Therefore, identifying these altered pathways may lead to effective prognostic biomarkers for improved treatment strategies.

Recent studies indicate that cholesterol, a critical component of the plasma membrane and lipid rafts, plays a significant role in tumorigenesis by supporting cancer cell adhesion and migration resulting in metastasis [3, 4]. Indeed, increased levels of cholesterol were observed in bone metastasis of prostate cancer [5]. Cells acquire cholesterol either from endogenous de novo synthesis or from the diet, via low-density lipoprotein (LDL) [6]. Excessive lipids and cholesterol in cancer cells are converted to triglycerides and fatty acid sterol esters (CE) and stored in lipid droplets [7]. Accumulation of intra-tumoral CE is known to alter cell signaling mechanisms leading to increased tumor proliferation, invasiveness and survival [8–10]. Therefore, inhibition of CE synthesis has been suggested as a potential anticancer therapeutic strategy [11, 12]. Cholesterol is esterified to CE by acyl-CoA cholesterol acyltransferase (ACAT1), also known as sterol O-acyltransferase (SOAT). ACAT1 is involved in maintaining appropriate levels of CE in non-tumor cells to support membrane stability. Abnormal ACAT1 expression and CE levels were found in cancer cells, including those of leukemia, glioma, prostate cancer, pancreatic cancer, breast cancer and colon cancer [13–17].

While the role of ACAT1/CE accumulation is being studied in various cancers [13–17], information regarding their contribution in EOC is scarce. We have recently reported increased levels of ACAT1 and CE in EOC cell lines compared to the primary ovarian epithelial cells (from normal ovaries), confirming ACAT1 mediated CE accumulation is a cancer-specific event [18]. ACAT1 inhibition and CE depletion have anti-tumor effects, as measured by apoptosis regulation, cisplatin sensitivity, and cell proliferation, migration and invasion [18]. These in vitro findings prompted us to investigate whether ACAT1 and CE effects occur in EOC tissue samples in order to extrapolate our observations in cell lines to clinical scenarios. This information is essential to assess the utility of ACAT1/CE as potential therapeutic targets for EOC.

The utility of tissue levels of ACAT1 and CE as prognostic markers for various cancers has been thoroughly researched [13–17, 19]; however, very few studies have investigated tumor ACAT1 and CE levels specifically in EOC and, to date, their levels in peritoneal fluid and plasma of EOC patients have not been studied. Consequently, we comprehensively assessed the levels of ACAT1 and CE in tumor tissue, peritoneal fluid and plasma from EOC patients (compared with normal ovary or benign pelvic mass samples) in order to determine the relationship between ACAT1/CE levels and various factors including malignancy, tumor aggressiveness (ki67 expression), body mass index (BMI) and various comorbidities. Possible correlations of ACAT1/CE levels between plasma, peritoneal fluid and ovarian/tumor tissue were also assessed to evaluate their diagnostic potential.

## Methods

### Ethic statement, standard protocol approvals, registrations and patient consents

The Springfield Committee for Research Involving Human Subjects approved this pilot study under protocols 12–656 and 16–493. Patients with a pelvic/adnexal mass or suspected ovarian cancer who were scheduled for a hysterectomy, oophorectomy, bilateral salpingo-oophorectomy (BSO), hysterectomy/BSO, tumor debulking and/or staging performed laparoscopically or via laparotomy were enrolled at the Division of Gynecological Oncology, Department of Obstetrics & Gynecology, Southern Illinois University School of Medicine. Patients with normal ovaries, scheduled to undergo the aforementioned procedures for the management of other gynecological diagnoses (e.g., pelvic prolapse) were enrolled within the divisions of General Gynecology and Urogynecology. Exclusion criteria included a previous malignancy, chemotherapy or radiation therapy prior to surgery. Eligible patients (age ≥ 30 years) were enrolled upon informed consent obtained during their preoperative visit. All sample collections were performed on the day of surgery. After surgery, subjects were grouped into three study cohorts based on their final pathological diagnosis: 1) subjects with a confirmed diagnosis of EOC (“EOC” group; *N* = 31); 2) those diagnosed with a benign pelvic mass (“BPM” group; *N* = 12) and 3) subjects with normal ovaries (“normal” group; *N* = 8). In order to assess the ability of the biomarkers to differentiate non-malignant from malignant EOC tumors, data from the BPM and normal groups were pooled together into what is labeled a “non-malignant” group (*N* = 20), for comparison against the malignant (EOC) group. Relevant clinical information was collected from electronic health records, including: age, menopausal status, cancer diagnosis, FIGO stage/grade (confirmed by independent pathologists), and presence of comorbidities such as obesity, dyslipidemia, diabetes, hypertension and hypothyroidism. Table 1 summarizes the clinical and pathological characteristics of the study population.

**Table 1:** Clinical and pathological characteristics of samples

Parameters	Normal	BPM	EOC
Sample Size N^a^ (%)	8 (14.5)	17 (30.5)	31 (55)
Age - Median (min - max)	61.5 (52–75)	58.0 (38–81)	60.0 (48–82)
BMI - Median (min - max)	30.7 (24.53–30.98)	28.00 (19.11–52.00)	29.3 (19.53–47.11)
Premenopausal N (%)^b^	2 (25)	7 (41)	2 (6)
Postmenopausal N (%)^b^	6 (75)	10 (59)	29 (94)
Obesity N (%)^b^	3 (38)	3 (18)	9 (29)
Diabetes N (%)^b^	3 (38)	3 (18)	5 (17)
Hyperlipidimia N (%)^b^	5 (63)	3 (18)	13 (41)
Hypertension N (%)^b^	4 (50)	7 (41)	7 (22)
Hypothyroidism N (%)^b^	4 (50)	4 (24)	4 (13)
**FIGO stage - N (% of EOC)**			
Stage I		8 (25.8)	
Stage II		4 (12.9)	
Stage III		17 (54.8)	
Stage IV		2 (6.5)	
**Histotype - N (% of EOC)**			
Serous		24 (77.4)	
Mucinous		4 (12.9)	
Endometrioid		3 (9.7)	

*BMI* Body mass index, *FIGO* International Federation of Gynecology and Obstetrics, *BPM* Benign Pelvic Mass, *EOC* Epithelial ovarian cancer. Obesity is defined as BMI ≥ 30. ^a^ Indicates total number of patients eligible for the study. ^b^ Percentages are calculated within each study group

### Peripheral blood, peritoneal fluid and tumor tissue sample collection

Peripheral blood was collected into sodium heparin tubes just prior to surgery. Peritoneal fluid was collected during the surgical procedure as previously described [20]. Briefly, after aspiration of ascites (if present), ~ 300 mL of isotonic saline (0.9% NaCl) were infused into the peritoneal cavity. This fluid was re-aspirated and refrigerated until further processing was complete. Plasma and peritoneal fluid samples were centrifuged at 1500 r/min for 10 min and stored at − 80 °C before being tested. Fragments (≥ 1cm^3^) of fresh tissue without necrotic areas were collected from the ovaries of subjects immediately after the oophorectomy was completed. A macro-dissection of the tissue samples was performed to remove fatty tissue and exclusively collect tumor, benign or normal ovarian tissue. These specimens were flash frozen in liquid nitrogen and stored at − 80 °C until analyzed.

### ACAT1 protein quantification by enzyme-linked immunosorbent assay (ELISA)

The ELISA Kit (Human) from Mybiosource (San Diego, CA, USA) was utilized to determine ACAT1 protein concentrations in plasma and peritoneal fluid as well as tissue lysates according to the manufacturer’s protocol. Tissue samples were homogenized in lysis buffer (1% Triton X-100, 150 mM NaCl, 50 mM Tris-HCl, 1 mM EGTA, 0.1% sodium dodecyl sulfate) supplemented with 1 mM PMSF and 1X complete protease inhibitor (A32955, ThermoFisher Scientific, MO, USA), and then sonicated. A bicinchoninic acid (BCA) protein assay kit (Bio-Rad, USA) was utilized to assess ACAT1 concentration. A Synergy H1MFD (Hybrid multimode) microplate reader (BioTek, VT, USA) was used to determine absorbance 450 nm and values were interpolated in a standard curve to calculate ACAT1 concentration (pg/mL).

### Lipid extraction and semi-quantitative analysis of CE, free cholesterol (FC) and total cholesterol (TC) in tissue samples

Tissue was extracted according to Bligh and Dyer method (1959) with modifications [21]. Briefly, 50 mg tissue was homogenized in 0.9 mL aqueous NaOH (0.1 M) and extracted with 1 mL methanol:chloroform (1:1). The extract was spun at 3000×g for 10 min at 15 °C. The methanol phase was retained, extracted with 1 mL chloroform and spun as indicated above. The chloroform phase, containing the lipids, was retained and allowed to evaporate at 22 °C to a final volume of 30 μL. CE and FC were partitioned by thin layer chromatography as previously described [22]. Briefly, 1-3 μL of each sample were spotted on the Silica thin layer chromatography (TLC) plates and developed with a heptane:diethylether:acetic acid (70:20:4) mixture. Plates were allowed to dry and stained in a solution of phosphomolybdic acid in ethanol (5% w/v) for 2 min, then developed at 120 °C for 30 min. This procedure yielded dark blue bands on a yellow background. The different concentrations of lipid standards (FC, cholesteryl palmitate, cholesteryl oleate) were run in parallel for identification and quantification of the sample bands. TLC plates were scanned on a GeneSys G: Box Chemi XT4 imager and signals were quantified using GeneTools version 4.3.9 software (Syngene, Cambridge, UK). The spots corresponding to CE and FC were densitometrically quantified against the standard curve of cholesterol palmitate and cholesterol, respectively, using a computing densitometer.

### Quantitative analysis of CE, FC and TC from plasma and peritoneal fluid

As described previously [18], we utilized the Total Cholesterol and Cholesteryl Ester Colorimetric Assay Kit (Biovision; Milpitas, CA, USA) for quantification of TC (cholesterol and CE), FC and CE from plasma and peritoneal fluid. Briefly, CE, FC and TC concentrations were determined in 50 μL aliquots of sample following the kit manufacturer instructions. The absorbance was measured at 570 nm using Synergy H1MFD (Hybrid multimode) microplate reader (BioTek, VT, USA). Concentrations of TC (mg/dL) were calculated by interpolation from a standard curve.

### Immunohistochemistry (IHC)

Immunohistochemical analysis for ACAT1 was performed on paraffin-embedded ovarian tissue section slides generated by the Springfield Memorial Hospital Laboratory as surgical pathology standard of care samples. We also purchased ovarian disease spectrum tissue microarray slides from US Biomax (OV1005b) for ACAT1 and Ki67 staining. IHC was performed per standard IHC protocol. Briefly, the slides were deparaffinized, rehydrated and heated in a citrate-based (pH 6.0) antigen retrieval solution from vector laboratories (H-3300) to unmask the antigenic sites. The slides were then immersed in 3% H_2_O_2_ solution for 10 min at room temperature to block the endogenous peroxidase and subsequently blocked with 10% goat serum and further incubated with appropriate primary antibodies (ACAT1 1:500 dilution, Ki67 1:500 dilution) for 1 h at room temperature. We used recombinant Anti-Ki67 (ab92742) and anti-SOAT 1/ACAT1 (ab39327) primary antibodies from Abcam (MA, USA). After the required washings, slides were incubated with their respective secondary antibodies for 10 min followed by 10 min incubation with streptavidin peroxidase. The antigen presence was revealed with 3.3′-diaminobenzidine (DAB) substrate (Abcam) and slides were counterstained with hematoxylin. To exclude any nonspecific staining of the secondary antibodies, negative controls were performed without the addition of any primary antibody. Additionally, IgG isotype controls (Rabbit IgG, monoclonal, ab172730 and Rabbit IgG, polyclonal, ab171870)) were also used as negative controls to determine background staining during method optimization studies. Representative images were taken with an inverted microscope (Olympus H4–100, CCD camera) and 20× objective. Five images in each core were captured and 1 μm wide z-stacks acquired. The images were analyzed via ImageJ software (NIH). One slide per sample was stained with hematoxylin and eosin for pathological examination.

ACAT1 staining was detected in the cytoplasm of cells, consistent with its known endoplasmic reticulum location. ACAT1 total staining score (data not shown) is calculated by the formula:

total score = staining intensity score × staining positive rate score.

The staining intensity is scored as: 0 points (negative), 1 point (weak), 2 points (moderate), and 3 points (strong). The staining positive rate is scored based on the positive cells as: 0 points (negative), 1 point (1–25%), 2 points (26–50%), 3 points (51–75%), and 4 points (76–100%). A total score of 2–6 was considered positive, while a score of 0 or 1 was considered negative [23].

For nuclear protein Ki-67, the percentage of stained tumor cells was used to calculate the Ki-67 immunostaining index (LI). Ki-67 LI is considered high when > 50% immunoreactive cells are positive and low when 50% or less immunoreactive cells are positive [24].

### RNA extraction and cDNA synthesis

Total RNA from EOC tumors, benign pelvic masses and normal ovarian tissues were isolated using TRIzol Reagent (Invitrogen, Carlsbad, CA, USA). RNA yield and quality were assessed by spectrophotometry and then stored at − 80 °C until use. A total of 1 μg RNA from each sample was reverse transcribed into cDNA using the iScript cDNA synthesis kit (BIO-RAD, CA).

### Gene expression analyses by qRT-PCR

Quantitative real-time reverse transcriptase-polymerase chain reaction (qRT-PCR) was utilized to determine ACAT1 and Ki67 mRNA levels. ACAT1 and Ki67 specific primers were purchased from Integrated DNA Technologies, Inc. (Coralville, Iowa, USA). RPl4 was used as the housekeeping gene. Based on the literature [25–27], we tested 18 s rRNA, IPO8, RPL4, TBP, RPLPO, ACTB and GAPDH for application as housekeeping genes. We found that RPL4 and ACTB consistently exhibited the least variation in expression across all tissue samples (normal ovaries, benign masses, and malignant ovarian tumors); therefore, we used those as reference genes for normalization of target gene expression. As compared to ACTB, RPL4 showed a more stable expression, therefore we presented RPL4 normalized data in this study. Normalization of target gene with either of these two housekeeping genes revealed equivalent patterns.

Transcript analysis was done using PowerUP SYBR Green Master Mix (Applied Biosystems, CA). The qRT-PCR reaction system included PowerUP SYBR master mix 5.0 μl, 0.1 μl forward primer (10 μM), 0.1 μl reverse primer (10 μM), 1.0 μl cDNA and 3.8 μl RNase free dH_2_O. All qRT-PCR reactions were performed under the following conditions: 50 °C for 2 min, 95 °C for 2 min, followed by 40 cycles of denaturation at 95 °C for 15 s, annealing at 55 °C for 15 s and extension at 72 °C for 1 min. Applied Biosystems 7500 Real Time PCR System (Applied Biosystems, CA) was used for qRT-PCR analysis. The thermal expression levels were measured in triplicate. The threshold cycle (Ct) values were normalized to the housekeeping gene and relative mRNA expression was determined using the ΔΔCt method [28].

### Statistical analysis

Descriptive statistics were used to characterize the samples and to describe the clinical/pathological variables and comorbidities. Data are presented as frequencies (percentages) for categorical variables, and medians (interquartile ranges) for continuous variables. Continuous variables were compared between non-malignant and EOC group using Mann Whitney non-parametric t test. Differences were considered significant if *p*-value < 0.05. To further compare between normal, BPM and EOC groups, we used Krusall-Wallis non-parametric ANOVA with Dunn’s multiple comparison post-hoc test (when appropriate). Correlations between different continuous variables were performed with Spearman’s rank test. To analyze the influence of confounder variables (BMI and comorbidities) on the associations between the ACAT1/CE content and malignancy (EOC), logistic regressions were performed. Model 1 is unadjusted model whereas model 2 adjusted predictor variables with different confounder variables individually. Differences were considered statistically significant when adjusted *p*-values < 0.05. All statistical analyses were performed using GraphPad Prism 7.04 and SPSS statistical software (SPSS Inc., Chicago, IL).

## Results

### Increased ACAT1 mRNA expression and protein production in EOC tumor tissues

**Fig. 1:**
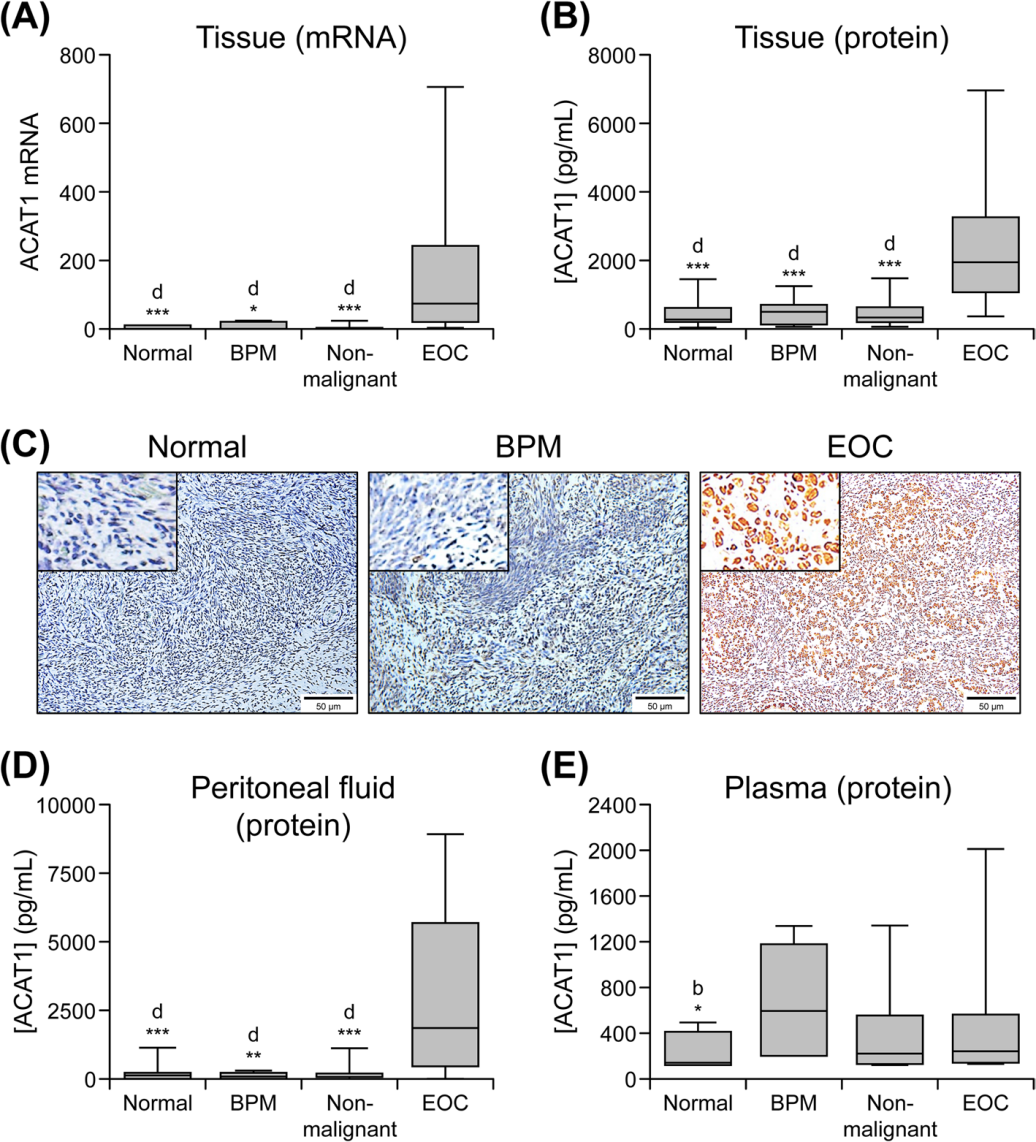
ACAT1 mRNA and protein levels in biological samples. Samples were collected from non-malignant (i.e., subjects with normal ovaries [Normal] and benign pelvic mass [BPM]) and ovarian cancer (EOC) patients. Box plots show medians (interquartile ranges) and whiskers (the minimum and maximum values). “d” indicates statistically significant difference compared to the EOC cohort. *: *p* < .05; **: *p* < .001; ***: *p* < .0001. (**a**) ACAT1 mRNA transcript levels assessed in ovarian tissue by qRT-PCR (non-malignant, *n* = 11; normal *n* = 7; BPM *n* = 4 and EOC subjects, *n* = 14). (**b**) ACAT1 protein expression levels in tissue assessed via ELISA (non-malignant, *n* = 19; normal, *n* = 12; BPM, *n* = 7 and EOC subjects, *n* = 14; triplicate experiments). (**c**) ACAT1 expression shown by DAB staining (brown) in human normal ovary, BMP and advanced stage EOC tumor samples. Representative images were taken with an inverted microscope (Olympus H4–100, CCD camera) and a 20X objective. Insets show images photographed with a 40X objective; *n* = number of samples. (**d**) ACAT1 protein expression levels (ELISA; triplicate experiments) in peritoneal fluid (non-malignant, *n* = 20; normal *n* = 8; BPM *n* = 12 and EOC, *n* = 27). (**e**) ACAT1 protein expression levels (ELISA, triplicate experiments) in plasma (non-malignant, *n* = 13; normal, *n* = 8; BPM *n* = 5; EOC, *n* = 16)

As shown in Fig. 1a, qRT-PCR analysis showed that ACAT1 mRNA levels were significantly higher (*p* < 0.001) in the tumor tissue of women with EOC (*n* = 14) versus those measured in ovarian tissue from the combined non-malignant group (*n* = 11). We further compared ACAT1 mRNA transcript levels in EOC versus normal (*n* = 7) and BPM (*n* = 4) groups separately in order to assess the ability of these markers to differentiate EOC tumors from benign masses or normal ovarian tissue. ACAT1 mRNA expression levels were significantly higher in EOC tissue compared to normal ovarian tissue (*p* = 0.0006) and benign masses (*p* = 0.0264).

Consistent with mRNA expression, ACAT1 protein levels were significantly higher (*p* < 0.001) in tumor tissue of women with EOC (*n* = 14) than those in samples collected from the non-malignant group (*n* = 19; Fig. 1b). Moreover, ACAT1 protein levels in EOC tumor tissue were significantly higher compared to pelvic masses (*n* = 7; *p* = 0.0002) and normal ovaries (*n* = 19; *p* = 0.0085) separately. No differences were observed between the normal and BPM groups with respect to both ACAT1 mRNA and protein. IHC analysis confirmed increased expression of ACAT1 protein in tumor tissue from the EOC group (evidenced by increased DAB staining) compared to normal or BPM tissues (Fig. 1c). Spearman correlation analysis showed significant positive correlation between ACAT1 protein and ACAT1 mRNA levels in tissue (*n* = 23, r = 0.72, *p* = 0.0002).

### Increased ACAT1 (protein) levels in peritoneal fluid of EOC patients

Figure 1d indicates that ACAT1 protein levels were significantly higher (*p* < 0.001) in the peritoneal fluid of women from the EOC group (*n* = 27) than those in the combined non-malignant group (*n* = 20). We also observed significantly higher levels of ACAT1 in the peritoneal fluid of women with EOC when compared to those in the normal group (*n* = 8; *p* < 0.0027) and BPM group (*n* = 12; *p* < 0.0001) separately. No significant differences were observed between normal and BPM groups (*p* > 0.999). Additionally, peritoneal fluid ACAT1 protein levels correlated positively with tissue ACAT1 mRNA (*n* = 23, Spearman *r* = 0.611, *p* = 0.002).

### Plasma ACAT1 level in EOC and non-malignant groups

Unlike tissue and peritoneal fluid ACAT1 levels, plasma ACAT1 levels did not differ significantly (*p* > 0.05) between the EOC (*n* = 16) and non-malignant group (*n* = 13; Fig. 1e). Moreover, plasma ACAT1 levels did not differ significantly between EOC, BPM (*n* = 5) and normal (*n* = 8) groups when compared separately (p > 0.05). No correlation was found between plasma ACAT1 protein concentrations and tissue ACAT1 mRNA transcript levels (*n* = 23, Spearman *r* = 0.052, *p* = 0.813).

### Correlation between ACAT1 protein concentrations in tissue, peritoneal fluid and plasma

**Fig. 2:**
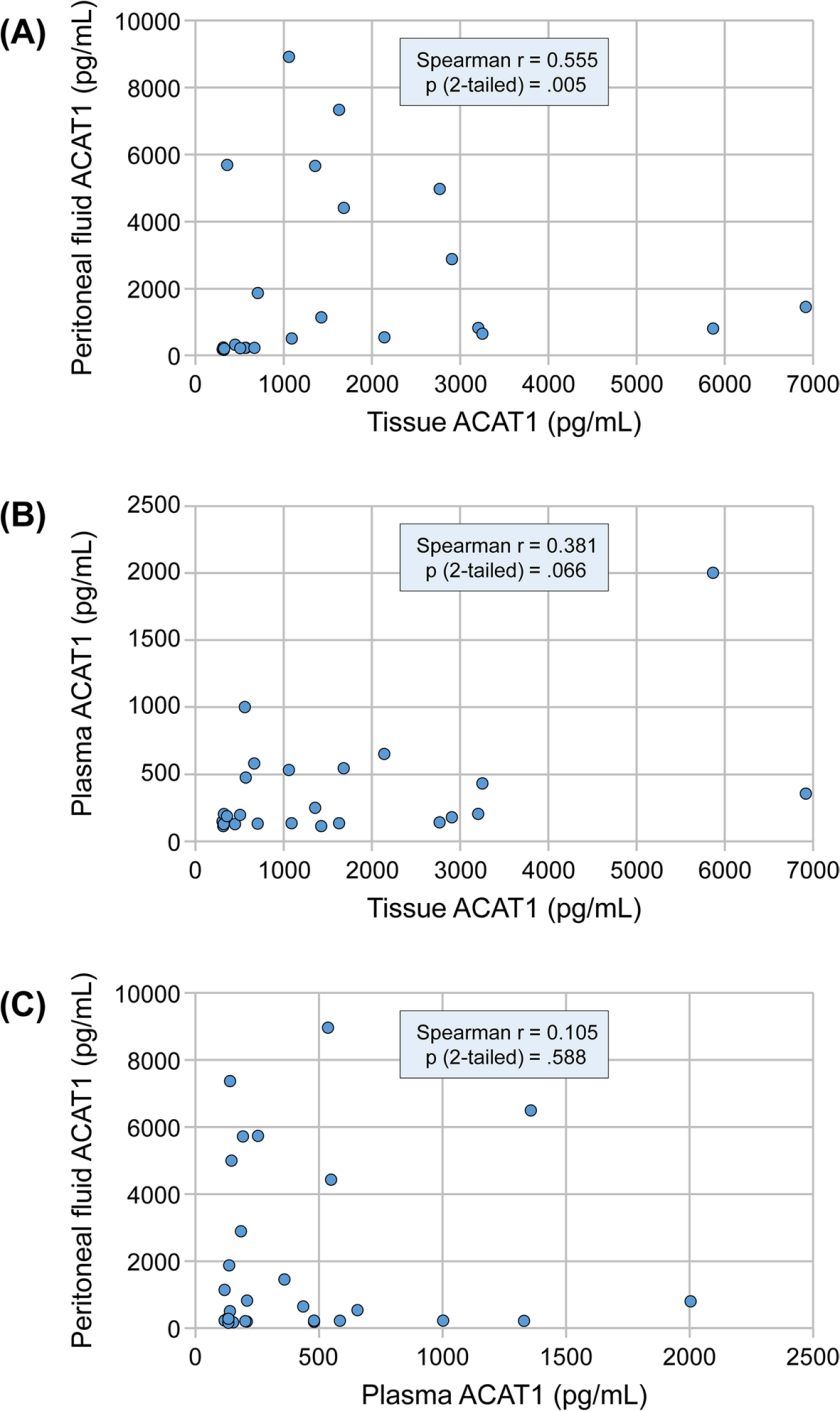
Spearman correlations of ACAT1 protein levels between tissue, peritoneal fluid and plasma. (**a**) Correlation between peritoneal fluid and tissue (*n* = 23). (**b**) Correlation plasma and tissue (*n* = 23). (**c**) Correlation between peritoneal fluid and plasma (*n* = 29)

In order to determine whether peritoneal fluid and plasma ACAT1 levels can predict tumor ACAT1 status, we assessed the correlation between tissue, peritoneal fluid and plasma ACAT1 protein levels. Figure 2a shows that peritoneal fluid levels of ACAT1 positively correlated with ACAT1 levels in tissue (*n* = 23, Spearman *r* = 0.555, *p* = 0.005); however, plasma ACAT1 levels did not significantly correlate with either tissue (Fig. 2b, *n* = 23, Spearman *r* = 0.381, *p* = 0.066) or peritoneal fluid ACAT1 levels (Fig. 2c, *n* = 29, Spearman *r* = 0.105, *p* = 0.588).

### CE, TC and FC levels in peritoneal fluid and plasma

**Fig. 3:**
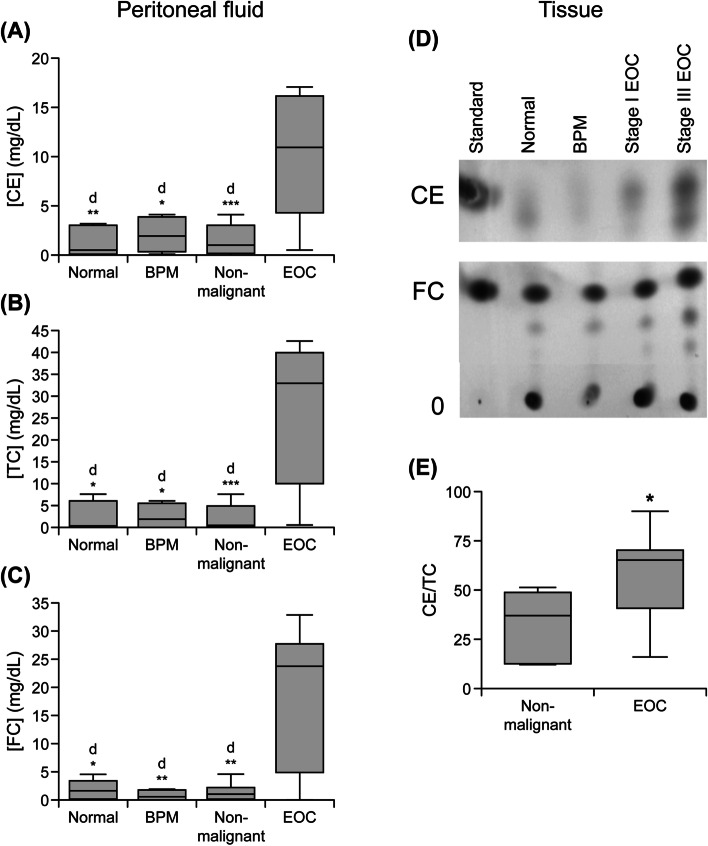
Lipid profiles in biological samples. Samples were collected from non-malignant (subjects with normal ovaries [Normal] and benign pelvic masses [BPM]) and ovarian cancer (EOC) patients. Box plots show medians (interquartile ranges) and whiskers (the minimum and maximum values). “d” indicates statistically significant difference compared to EOC *: *p* < .05; **: *p* < .001; ***: *p* < .0001. (**a**-**c**) Cholesteryl ester (CE), total cholesterol (TC) and free cholesterol (FC) levels in peritoneal fluid (non-malignant, *n* = 9; normal, *n* = 5; BPM *n* = 4; EOC, *n* = 15). Experiments were performed in triplicate. (**d**-**e**) Thin layer chromatography analysis of lipids in tissue samples. (**d**) Lipids were extracted from tissue samples, resolved by thin layer chromatography, stained and quantified. Lipids were identified FS or CE by comparison to standards. Representative results of normal ovary, BPM, EOC stage I and EOC stage III tissue lipids are shown. (**e**) Percent of CE to TC (non-malignant, *n* = 7; normal, *n* = 2; BPM *n* = 5; EOC, *n* = 12). Asterisks indicate statistically significant difference between non-malignant and EOC cohorts (*: *p* < .05)

As shown in Fig. 3a-c, CE, TC and FC levels were significantly higher (*p* < 0.001) in the peritoneal fluid of women with EOC (*n* = 15) compared to those from the non-malignant group (*n* = 9). These factors were also elevated in the EOC group versus BPM (*n* = 4) and normal (*n* = 5) groups separately (*p* < 0.05). No significant differences were observed between normal and BPM groups for any of these analytes. There was a strong positive correlation between TC, FC and CE levels in peritoneal fluid (*n* = 24, Spearman *r* = 0.80; *p* < 0.0001).

Similar to our observations of ACAT1 protein concentrations in plasma samples, levels of TC, FC and CE in plasma did not differ significantly (*p* > 0.05) between the EOC (*n* = 13) and non-malignant group (*n* = 13, data not shown).

### Tissue TC, FC and CE levels

To eliminate the variations observed during lipid extraction, thin layer chromatographic analysis of lipids extracted from tissue samples is shown as percent of CE to TC. The overall percent of CE to TC (CE + FC) was significantly higher (*p* = 0.022) in tissue extracts of EOC (*n* = 12) versus lipids extracted from tissue samples collected from non-malignant group (*n* = 7; 3d-e). The overall percent of CE was also significantly higher in tissue extracts of EOC when compared to the BMP (*n* = 5; *p* = 0.048) and normal (*n* = 2; *p* = 0.027) groups separately.

### Correlation between ACAT1 and CE levels in ovarian tissue, plasma and peritoneal fluid

Table 2 shows Spearman correlation analysis indicating significant correlation between ACAT1 protein and CE levels in tissue (*n* = 19, *r* = 0.552, *p* = 0.044) and peritoneal fluid (*n* = 24, *r* = 0.594, *p* = 0.007). Interestingly, a strong correlation was also observed between tissue ACAT1 expression and peritoneal fluid CE levels (*n* = 24, *r* = 0.787, *p* = 0.002) or peritoneal fluid ACAT1 and tissue CE levels (*n* = 19, *r* = 0.574, *p* = 0.035). This may imply that tissue ACAT1 mediates secretion of CE into peritoneal fluid; therefore assessment of CE from peritoneal fluid may be indicative ACAT1 levels in tissue and tumor aggressiveness. However, plasma ACAT1 protein did not show any correlation with CE levels in tissue (*n* = 19), peritoneal fluid (*n* = 24) or plasma (*n* = 24).

**Table 2:** Correlation between CE and ACAT1 levels in tissue, peritoneal fluid and plasma

		Spearman R values	*p*-Values
ACAT1 Protein (**Tissue**)	versus CE^a^ (*n* = 19)	0.552	0.044
	versus CE^b^ (*n* = 24)	0.787	0.002
	versus CE^c^ (*n* = 26)	−0.243	0.347
ACAT1 Protein (**Peritoneal Fluid**)	versus CE^a^ (*n* = 19)	0.574	0.035
	versus CE^b^ (*n* = 24)	0.594	0.007
	versus CE^c^ (*n* = 24)	0.231	0.313
ACAT1 Protein (**Plasma**)	versus CE^a^ (*n* = 19)	0.134	0.648
	versus CE^b^ (*n* = 24)	0.236	0.330
	versus CE^c^ (*n* = 26)	−0.361	0.108

*CE* Cholesterol ester, *ACAT*1 acyl-CoA cholesterol acyltransferase, ^a^ tissue, ^b^ peritoneal fluid, ^c^ plasma, *n* = number of samples

### Correlation between Ki67 expression and ACAT1 (protein, mRNA), CE, TC and FC levels

**Fig. 4:**
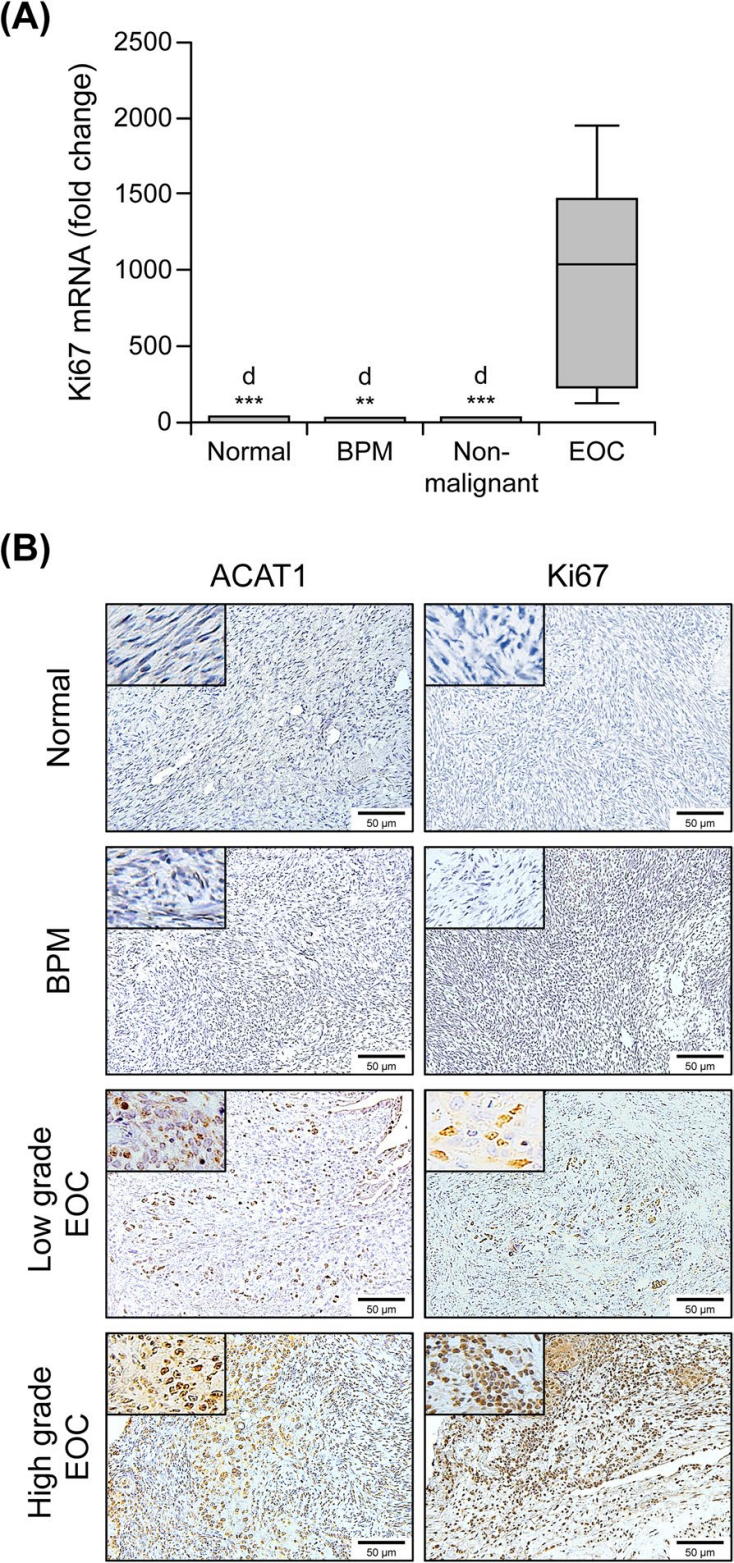
Ki67 expression in tissue samples. (**a**) Ki67 mRNA transcript levels assessed via qRT PCR in non-malignant tissue (*n* = 12, of which 7 had normal ovaries and 5 had benign pelvic masses) and ovarian cancer (EOC, *n* = 14) tissue samples. Box plots show medians (interquartile ranges) and whiskers (the minimum and maximum values). “d” indicates statistically significant difference compared to EOC (*: *p* < .05; **: *p* < .001; ***: *p* < .0001). (**b**) Ki67 protein immunostaining in tissue. Ki67 expression shown by DAB staining (brown) in normal human ovary, benign pelvic masses (BMP), low grade EOC and high grade EOC tumor samples. Representative images were taken with an inverted microscope (Olympus H4–100, CCD camera) and a 20X objective. Insets show images photographed with a 40X objective

As shown in Fig. 4a, Ki67 mRNA transcript levels in EOC tissue (*n* = 14) are significantly elevated (*p* < 0.0001) compared to those in non-malignant tissues (*n* = 12). This increased Ki67 mRNA expression in EOC tissue was also observed when compared with normal (*n* = 7; *p* = 0.0007) and BPM (*n* = 5; *p* = 0.0036) groups separately.

Spearman correlation analysis (Table 3) indicated a significant positive correlation between tissue Ki67 mRNA and tissue ACAT1 protein (*r* = 0.755, *p* < 0.0001), tissue ACAT1 mRNA (*n* = 25, *r* = 0.703, *p* < 0.0001), and peritoneal fluid ACAT1 protein (*n* = 25, *r* = 0.542, *p* < 0.0014). Similarly, a positive association was seen between Ki67 and CE levels assessed from tissue (*n* = 19, *r* = 0.561, *p* = 0.0322) and peritoneal fluid (*n* = 24, *r* = 0.559, *p* = 0.021). We also observed a significant positive correlation between Ki67 and TC, FC levels from peritoneal fluid (*n* = 24, *r* = 0.612, *p* = 0.010 and *r* = 0.568, *p* = 0.019, respectively). Interestingly, no correlation was observed between Ki67 and any of the analytes measured in plasma (*n* = 25, *p* < 0.05).

**Table 3:** Spearman’s rank coefficient analyses of Ki67 correlation with ACAT1, TC, FC and CE levels

Ki67 (mRNA)	Spearman r	***p*** value	Significance
versus Stage (*n* = 26)	0.352	0.036	*
versus Tissue ACAT1 protein (*n* = 26)	0.755	< 0.0001	***
versus Tissue ACAT1 mRNA (*n* = 25)	0.703	< 0.0001	***
versus Peritoneal Fluid ACAT1 protein (*n* = 26)	0.542	0.001	**
versus Plasma ACAT1 protein (*n* = 26)	0.109	0.604	NS
versus Tissue CE (*n* = 19)	0.561	0.032	*
versus Peritoneal Fluid CE (*n* = 24)	0.559	0.021	*
versus Peritoneal Fluid TC (*n* = 24)	0.612	0.010	*
versus Peritoneal Fluid FC (*n* = 24)	0.568	0.019	*
versus Plasma CE (*n* = 26)	−0.294	0.251	NS
versus Plasma TC (*n* = 26)	0.093	0.723	NS
versus Plasma FC (*n* = 26)	0.191	0.461	NS

*ACAT*1 acyl-CoA cholesterol acyltransferase, *CE* cholesterol ester, *TC* total cholesterol, *FC* free cholesterol, *n* number of samples, *NS* Non significant; *: *p* < .05; **: *p* < .001; ***: *p* < .0001

Additionally, tissue sections exhibiting higher ACAT1 immunostaining also had higher Ki67 expression (Fig. 4b). Furthermore, the highest levels of ACAT1 immunostaining were observed in samples of advanced stage and high grade EOC tumors.

### Assessment of ACAT1 and CE as diagnostic markers for EOC

Receiver operating characteristic (ROC) curve analysis was performed to assess diagnostic potential of ACAT1 and CE in plasma, peritoneal fluid and tissue and to define optimal cut-off levels for ACAT1 and CE in the diagnosis of EOC. As shown in Table 4, assessing these markers in peritoneal fluid samples has better diagnostic power as compared to plasma levels. Peritoneal fluid ACAT1 level has the highest area under the curve (AUC) of ROC (0.944) followed by CE (0.913), TC (0.902) and FC (0.879). Peritoneal fluid ACAT1 level had a sensitivity of 93% and a specificity of 95% at a cut off concentration of > 270.08 pg/ml. Similarly, peritoneal fluid CE concentration at > 3.21 mg/dL had a specificity of 92% and a sensitivity of 89%. Moreover, tissue ACAT1 protein concentration had an AUC of 0.940. In contrast, plasma levels of ACAT1 or CE are not ideal for diagnostic assessments.

**Table 4:** ROC analysis of ACAT1/CE/TC/FC in peritoneal fluid, plasma and tumor tissue

Source	Analyte (n)	ROC	*p*-value
Peritoneal Fluid	ACAT1 Protein (*n* = 47)	0.9444	< 0.0001
	Cholesterol ester (*n* = 24)	0.9125	0.0011
	Total cholesterol (*n* = 24)	0.9015	0.0011
	Free cholesterol (*n* = 24)	0.8788	0.0021
Tissue	ACAT1 Protein (*n* = 33)	0.9398	< 0.0001
	ACAT1 mRNA (*n* = 25)	0.9805	< 0.0001
Plasma	ACAT1 Protein (*n* = 29)	0.621	NS
	Cholesterol ester (*n* = 26)	0.534	NS
	Total cholesterol (*n* = 26)	0.504	NS
	Free cholesterol (*n* = 26)	0.500	NS

*ACAT*1 acyl-CoA cholesterol acyltransferase, *CE* cholesterol ester, *TC* total cholesterol, *FC* free cholesterol, *NS* Non significant, *n* number of samples

### Assessment of the potential influence of comorbidities on the association of ACAT1/CE with EOC

Comorbidities such as obesity, hyperlipidemia, diabetes, hypertension and hyperthyroidism are known to alter the cholesterol metabolism; therefore, we analyzed the influence of BMI and comorbidities on the associations between ACAT1 or CE levels and EOC. Logistic regressions were adjusted for these co-variables (Model 2). We observed that the association of ACAT1 or CE levels with EOC remained statistically significant even when adjusted for these co-variables (*p* < 0.05; Table 5).

**Table 5:** Association between ACAT1 and CE levels with comorbidities

Model 1 (unadjusted)				Model 2 (adjusted by various comorbidities)			
	β	Standardized β (CI)	*p*-value		β	Standardized β (CI)	*p*-value
ACAT1-Peritoneal Fluid	0.006	1.006 (1.001–1.010)	0.010	BMI	0.006	1.006 (1.001–1.010)	0.010*
				Hyperlipidemia	0.006	1.006 (1.001–1.011)	0.010*
				Obesity	0.006	1.006 (1.001–1.010)	0.014*
				Diabetes	0.008	1.008 (1.002–1.013)	0.009*
				Hypertension	0.005	1.005 (1.001–1.010)	0.019*
				Hypothyroidism	0.006	1.006 (1.001–1.010)	0.014*
ACAT1-Tissue	0.003	1.003 (1.001–1.005)	0.009	BMI	0.003	1.003 (1.000–1.005)	0.018*
				Hyperlipidemia	0.003	1.003 (1.000–1.006)	0.024*
				Obesity	0.003	1.005 (1.000–1.005)	0.017*
				Diabetes	0.003	1.003 (1.000–1.005)	0.026*
				Hypertension	0.003	1.005 (1.000–1.005)	0.026*
				Hypothyroidism	0.003	1.006 (1.000–1.005)	0.023*
CE- Peritoneal Fluid	0.252	1.287 (1.026–1.614)	0.029	BMI	0.256	1.292 (1.028–1.623)	0.028*
				Hyperlipidemia	0.255	1.291 (1.015–1.641)	0.037*
				Obesity	0.254	1.289 (1.025–1.622)	0.030*
				Diabetes	0.288	1.334 (1.041–1.711)	0.023*
				Hypertension	0.269	1.309 (1.022–1.675)	0.033*
				Hypothyroidism	0.243	1.275 (1.019–1.595)	0.034*
CE-Tissue	0.009	1.009 (1.001–1.019)	0.041	BMI	0.010	1.010 (1.000–1.020)	0.049*
				Hyperlipidemia	0.011	1.011 (1.001–1.021)	0.040*
				Obesity	0.010	1.010 (1.000–1.021)	0.049*
				Diabetes	0.018	1.018 (0.999–1.038)	0.067
				Hypertension	0.011	1.011 (1.000–1.022)	0.043*
				Hypothyroidism	0.011	1.011 (1.001–1.021)	0.04*

*CI* confidence interval

### ACAT1 expression as prognostic biomarker in ovarian cancer

Due to the small sample size and non-availability of survival data (i.e., subjects lost to follow-up) for many of the subjects in our database, we did not perform survival data analysis for prognostic evidence. However, we used public databases such as the PrognoScan Online Platform and Kaplan-Meier Plotter Analysis to evaluate the prognostic value of ACAT1 expression for ovarian cancer prognosis. As shown in the supplementary material, both databases demonstrated the association of high ACAT1 expression with poor overall survival or unfavorable prognosis. Indeed, PrognoScan Online Platform showed a hazard ratio (HR) = 2.28 [1.36–3.82], *p* = 0.0019 (Supplementary Table 1), while Kaplan-Meier Plotter Analysis resulted in HR = 1.16 [1.02–1.32], *p* = 0.022 (Supplementary Fig. 1). However, as per The Cancer Genome Atlas (TCGA), when comparing groups based on best expression cut off or median values, no significant difference was observed in 5-year survival between high and low ACAT1 expression groups (Supplementary Fig. 2). As per the cBioportal database, genetic alteration of ACAT1 gene was reported in 4% of ovarian cancer patients (66 of 1713 patients), most of which are copy number amplifications. Kaplan-Meier estimate showed significant difference in overall survival between the ACAT1 altered group vs. the unaltered groups (Log Rank test *p*-value - 0.0114). Interestingly, favorable prognosis was observed with high ACAT1 expression. In contrast, when ACAT1 gene expression groups based on quartiles were compared, subjects with low levels of ACAT1 expression survived significantly longer than those with high levels of ACAT1 (*p* = 0.0233, Log Rank test), with a median survival time of 48 months for low-ACAT1 patients and 36 months for high-ACAT1 patients (supplementary Fig. 3). These clinical data suggest that ACAT1 expression may be a potential prognostic marker for ovarian cancer and warrants further investigation.

## Discussion

Ovarian cancer remains a challenging disease to treat [29, 30]; therefore, extensive research is needed to understand the disease complexity and to develop new treatment strategies.

Overexpression of ACAT1 followed by increased CE accumulation in lipid droplets has been reported in a variety of cancer types [13–16, 31, 32]. A correlation between ACAT1 overexpression and poor prognosis was confirmed in high-grade human prostate, pancreatic and breast cancers [16, 17, 19]. Although considerable research has been carried out with tumor tissues to establish ACAT1 as a potential new prognostic marker in these cancers [16, 17, 19], there have been very few studies examining ACAT1 expression and CE levels in tissue and plasma, and none within peritoneal fluid of EOC patients. Here, we systematically analyzed ACAT1 expression and CE levels in peritoneal fluid, plasma and tumor tissue in EOC patients and compared these to samples collected from patients with normal ovaries and/or benign pelvic masses. We also correlated the expression of these mediators with a marker of tumor aggressiveness (ki67 expression).

Many studies assessed ACAT1 protein expression in tumor tissues primarily by IHC; however, we comprehensively measured ACAT1 expression at both the protein and mRNA levels utilizing ELISA, IHC and qRT-PCR. The fact that ACAT1 expression was absent or minimal in benign masses and normal tissues and significantly higher in EOC samples indicates the importance of ACAT1 in EOC development; therefore, ACAT1 could be a potential tumor specific target. In plasma, ACAT1 levels did not differ significantly among the normal, BPM and EOC cohorts, suggesting that ACAT1 cannot be used as a non-invasive biomarker for diagnosis or prognosis. In contrast to our observations in plasma, but similar to our results in ovarian tumor tissue, ACAT1 protein levels from peritoneal fluid were significantly higher in women with EOC and very low in both normal and BPM cohorts. These results suggest that highly elevated ACAT1 levels in the peritoneal cavity of EOC patients may play an important role in the peritoneal dissemination (tumor invasion) and cancer aggression. Peritoneal fluid represents the local tumor microenvironment, consisting of both cellular and acellular components. Recent findings indicate that the tumor microenvironment contains pro-tumorigenic signals that contribute to enhanced invasiveness and chemoresistance [33, 34] resulting in disease progression. The concentration of these components varies between patients, according to their disease stage, grade and histological subtypes [33, 35]. These factors are either the cause or result of underlying disease. Indeed, our study decoded ACAT1 and CE as important components of the tumor microenvironment which may contribute to cancer aggressiveness and drug resistance.

Abnormal accumulation of CE has been reported in breast cancer, leukemia, glioma, pancreatic, renal and prostate cancers [13, 19]. Similarly, we observed a significant increase in CE, TC and FC levels in the peritoneal fluid and tumor tissue of EOC patients. Previous studies showed that cholesterol is significantly elevated in ascites and could be used as a marker for malignant ascites [36, 37]. Cholesterol is required for cellular proliferation through specific interactions with major signaling pathways [16, 38]. However, excess cellular cholesterol is toxic, and conversion of cholesterol to CE via ACAT1 may be one of the mechanisms to avoid toxicity, as well as to escape feedback inhibition and maintain a high metabolic activity needed for disease progression [39, 40]. Supporting this hypothesis, a significant positive correlation was observed between ACAT1 and CE levels in both peritoneal fluid and tissue.

ACAT1 and CE-rich tumors were associated with higher aggressive potential and poor survival in many cancers [19]. Consistent with these reports, we observed a significant positive correlation between ACAT1 (protein and mRNA), CE levels and Ki67. Ki67 is an established tumor proliferation marker known to predict disease outcome in many human malignancies [41]. Many studies demonstrated a positive relationship between high proliferation rates and poor survival or increased recurrence [41, 42]. The mechanism(s) underlying the relation between CE accumulation and cancer aggressiveness has not been precisely established. ACAT1 mediated accumulation of CE may alter cell signaling to promote tumor proliferation and survival [8–10]. Conversely, inhibition of CE generation may aid in the suppression of tumor proliferation. Indeed, inhibition of ACAT1 using pharmacologic (avasimibe) or genomic (ACAT1 shRNA) agents suppressed cancer cell proliferation, migration and invasion in vitro and in vivo as reported in colon, pancreas, prostate and EOC models [15, 17, 18, 43]*.* The molecular mechanism linking ACAT1 mediated inhibition of CE accumulation to tumor suppression is still not clear. Cholesterol esterification has been hypothesized to keep signaling pathways active and protect cells from FC toxicity. Therefore, inhibition of CE generation may disrupt intracellular cholesterol homeostasis and consequently inactivate Sterol Regulatory Element-Binding Protein 1 (SREBP1) leading to downregulation of SREBP1 regulated processes such as caveolin-1/MAPK activation, reduced LDLr expression and reduced LDLr mediated uptake of essential fatty acids, such as arachidonic acid (a proliferation factor in many cancers) [16, 17, 44]. Other possible signaling mechanisms include the downregulation of Wnt/β-catenin, pAkt and ERK1/2 pathways and inhibition of TLR4 (Toll-like receptor 4), all of which play significant roles in cancer cell proliferation and metastasis [45, 46].

In addition to promoting cancer aggressiveness, ACAT1/CE are also known to play an important role in drug resistance, which is the primary cause of EOC recurrence [47, 48]. Abnormal accumulation of ACAT1/CE may lead to resistance to drugs such as tamoxifen, gemcitabine, imatinib and cisplatin as shown in various in vitro and in vivo cancer models [18, 43, 49, 50]. Similarly, the clinical significance of cholesterol in EOC has been reported in recent studies, including the possible association between chemoresistance and elevated cholesterol levels in ascites [51, 52]. Therefore, inhibition of CE accumulation may enhance the sensitivity of cancer cells to drug treatments. Indeed Li et al., reported that the combination of avasimibe and gemcitabine reduced cell viability and tumor growth in pancreatic cancer cells [43]. Bandyopadhyay et al., showed that the synergistic effect of avasimibe and imatinib sensitized chronic myeloid leukemia cells to imatinib treatment [53].

Similarly, we have previously reported an increased sensitivity to cisplatin in ACAT1-inhibited cell lines of SKOV-3 and IGROV-1 compared to their uninhibited controls [18]. Although we do not know the specific mechanism(s) underlying this observation, ACAT1 inhibition and depletion of CE have been reported to inhibit PI3K/Akt, caveolin and MAPK pathways leading to increased sensitivity to drugs [16, 50]. Further research will be crucial to decode the relationship between cholesterol metabolism and chemotherapy drug resistance in EOC.

The search for precise and feasible EOC biomarkers and/or therapeutic targets is ongoing. Ovarian masses are diagnosed by conventional imaging methods such as transvaginal ultrasound; however, these methods cannot differentiate between malignant and benign lesions. Plasma/serum factors can be assessed noninvasively and are an ideal source of biomarkers. Although serum cancer antigen-125 (CA-125) is a widely accepted non-invasive biomarker for EOC, it lacks sensitivity and specificity, as it can be elevated in many benign diseases and other (non-ovarian) cancers [54, 55]. Therefore, additional non-invasive diagnostic and prognostic markers are still needed to assess disease status, progression and prognosis. In this study, we did not observe significant differences in plasma ACAT1 and CE levels between non-malignant and EOC groups. However, our data show that expression of these markers in peritoneal fluid correlated with the presence of EOC and tumor aggressiveness. Indeed, a significant correlation was observed between peritoneal fluid and tissue ACAT1 and CE levels; therefore, peritoneal fluid levels may predict the local tumor status and may serve as potential surrogate markers for diagnosis and aggressiveness in EOC patients.

During the course of tumor progression, EOC exhibits a wide range of morphological, clinical and genetic variations due to different cellular and molecular characteristics. Therefore, it is essential to decode the molecular profiles of EOC for patient-specific disease treatment (personalized medicine). Our study adds ACAT1 and CE to the current list of potential prognostic markers that may aid in predicting disease outcomes in patients with EOC. Further comprehensive prospective studies using large sample cohorts are needed to assess the correlation of plasma, peritoneal fluid and tumor ACAT1 with clinical and pathological features of patients.

## Conclusions

We demonstrated the prognostic relevance of ACAT1 and CE in tumor tissue and peritoneal fluid of EOC patients. We advanced the potential application of peritoneal fluid ACAT1 and CE levels as biomarkers for monitoring disease aggressiveness in patients with EOC. Further studies to establish altered lipid profiles in EOC are essential for identifying appropriate treatments for EOC.

## Supplementary Information

**Additional file 1: Supplementary Table 1.** Ovarian Cancer - PrognoscanOnline Platform Analysis. **Supplementary Figure 1**. Kaplan-Meier Plotter Analysis. Overall survival curves for SOAT1 (221561_at) were obtained for Ovarian Cancer patients divided into two groups as low-expression and high-expression based on the optimal cut-off value for survival. Information was obtained from the public database available at https://kmplot.com/analysis/. **Supplementary Figure 2**. The Cancer Genome Atlas (TCGA) Analysis. 5-year survival curves for Ovarian Cancer patients divided into two groups (low vs. high ACAT1 expression) based on the optimal cut-off value for survival. Information was obtained from the public database available at https://www.proteinatlas.org. **Supplementary Figure 3**. cBioPortaldatabase analysis. Overall survival curves for Ovarian Cancer patients divided into different ACAT1 gene expression groups based on quartiles. Information was obtained from the public database available at https://www.cbioportal.org.

## Data Availability

The datasets used and/or analyzed during the current study are available from the corresponding author on reasonable request.

## Abbreviations

ACAT1: Acyl-CoA cholesterol acyltransferase-1
CE: Cholesterol esterified with fatty acids
EOC: Epithelial ovarian cancer
ROC: Receiver operating characteristic
qRT-PCR: Quantitative real-time polymerase chain reaction
LDL: Low-density lipoprotein
LDs: Lipid droplets
BPM: Benign pelvic mass
ELISA: Enzyme-linked immunosorbent assay
FC: Free cholesterol
TC: Total cholesterol
TLC: Thin layer chromatography
IHC: Immunohistochemistry
BMI: Body mass index
AUC: Area under the curve
