# Supplementary material for: Assessment of the diagnostic and prognostic relevance of ACAT1 and CE levels in plasma, peritoneal fluid and tumor tissue of epithelial ovarian cancer patients - a pilot study
Source: BMC Cancer. 2022 Apr 10;22:387. doi: 10.1186/s12885-022-09476-6 (PMC8994887; doi:10.1186/s12885-022-09476-6)
Supplement: Supplementary file 1 — Additional file 1: Supplementary Table 1. Ovarian Cancer - PrognoscanOnline Platform Analysis. Supplementary Figure 1. Kaplan-Meier Plotter Analysis. Overall survival curves for SOAT1 (221561_at) were obtained for Ovarian Cancer patients divided into two groups as low-expression and high-expression based on the optimal cut-off value for survival. Information was obtained from the public database available at https://kmplot.com/analysis/. Supplementary Figure 2. The Cancer Genome Atlas (TCGA) Analysis. 5-year survival curves for Ovarian Cancer patients divided into two groups (low vs. high ACAT1 expression) based on the optimal cut-off value for survival. Information was obtained from the public database available at https://www.proteinatlas.org. Supplementary Figure 3. cBioPortaldatabase analysis. Overall survival curves for Ovarian Cancer patients divided into different ACAT1 gene expression groups based on quartiles. Information was obtained from the public database available at https://www.cbioportal.org. [file 12885_2022_9476_MOESM1_ESM.pdf]

Supplementary Table 1: Ovarian Cancer - Prognoscan Online Platform Analysis

Cohort	Contributor	Array type	Probe ID	N	COX p-value	HR [95% CI]
Duke	Bild	HG-U133A	221561_at	133	0.0145[a]	0.51 [0.29 - 0.87]
Milan (1992-2003)	Marchini	G4100A	12236	81	0.0119[a]	2.25 [1.20 - 4.22]
MSKCC (1990-2003)	Bonome	HG-U133_Plus_2	221561_at	185	0.0019[b]	2.28 [1.36 - 3.82]
MSKCC (1990-2003)	Bonome	HG-U133_Plus_2	221561_at	185	0.0216[a]	1.96 [1.10 - 3.47]

a: overall survival; b: disease free survival

Supplementary Figure 1: Kaplan-Meier Plotter Analysis. ***Overall survival*** curves for SOAT1 (221561_at) were obtained for Ovarian Cancer patients divided into two groups as low-expression and high expression based on the optimal cut-off value for survival. Information was obtained from the public database available at https://kmplot.com/analysis/

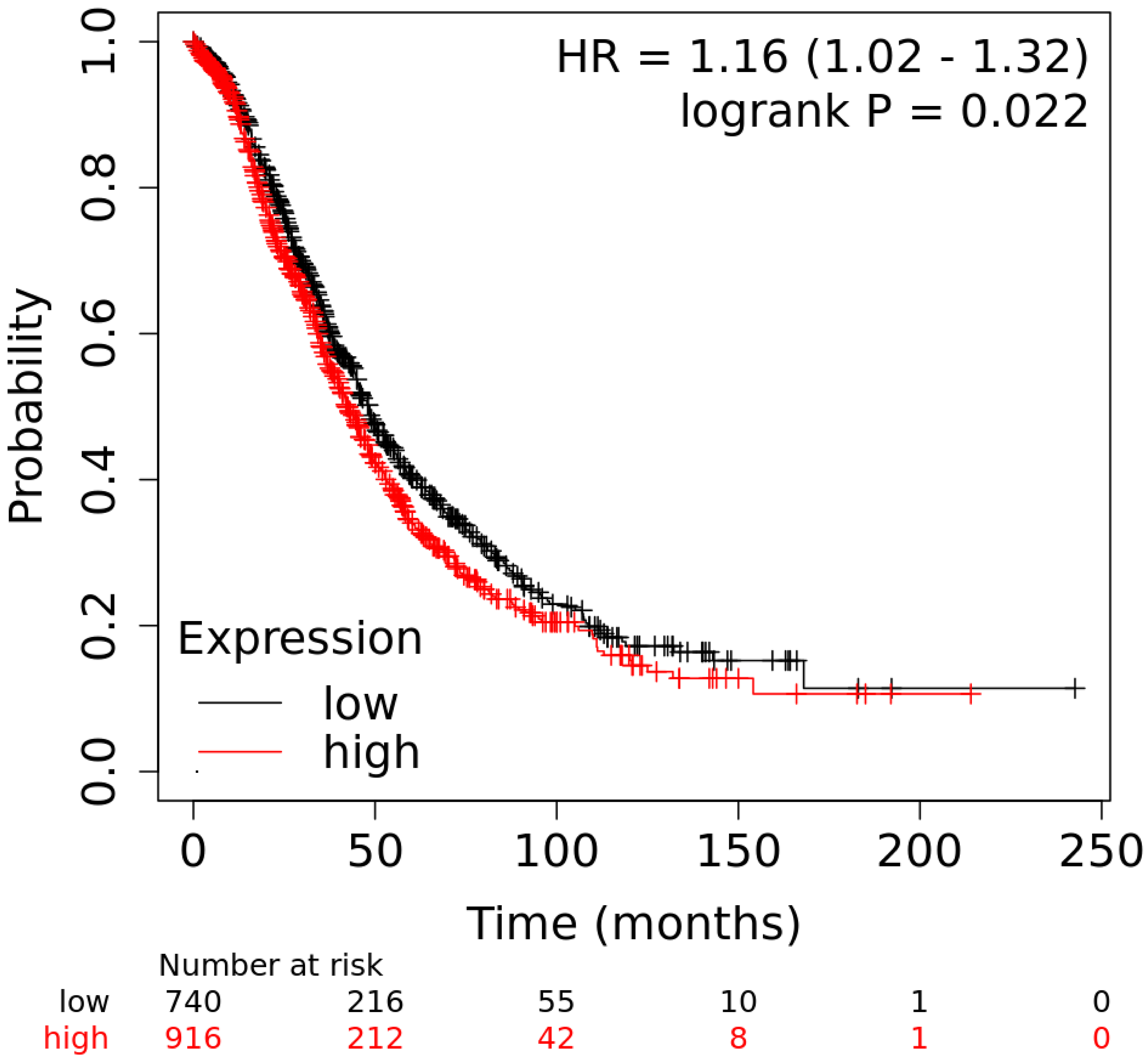

Supplementary Figure 2: The Cancer Genome Atlas (TCGA) Analysis. ***5-year survival*** curves for Ovarian Cancer patients divided into two groups (low vs. high ACAT1 expression) based on the optimal cut-off value for survival. Information was obtained from the public database available at https://www.proteinatlas.org

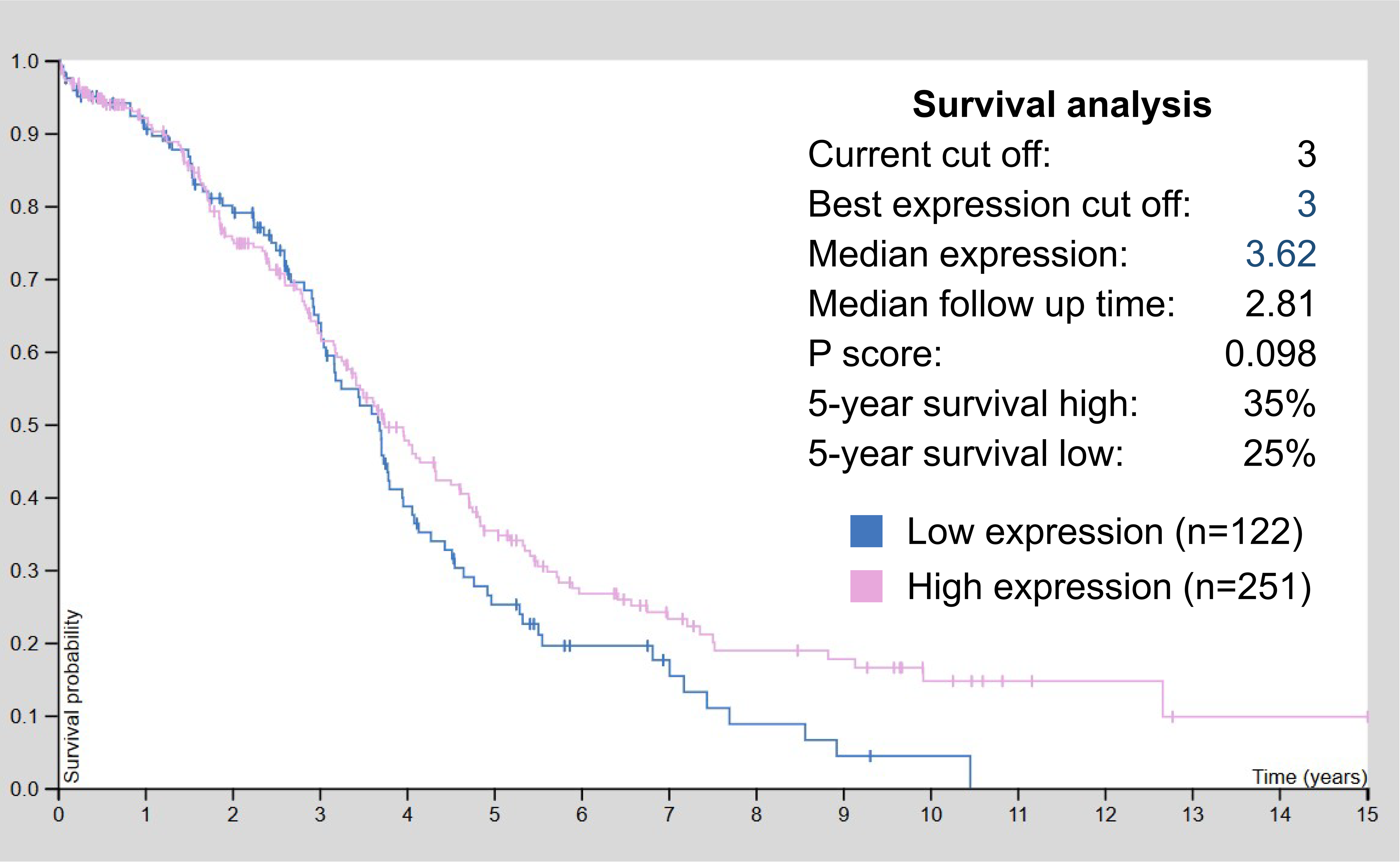

Supplementary Figure 3: cBioPortal database analysis. ***Overall survival*** curves for Ovarian Cancer patients divided into different ACAT1 gene expression groups based on quartiles. Information was obtained from the public database available at https://www.cbioportal.org

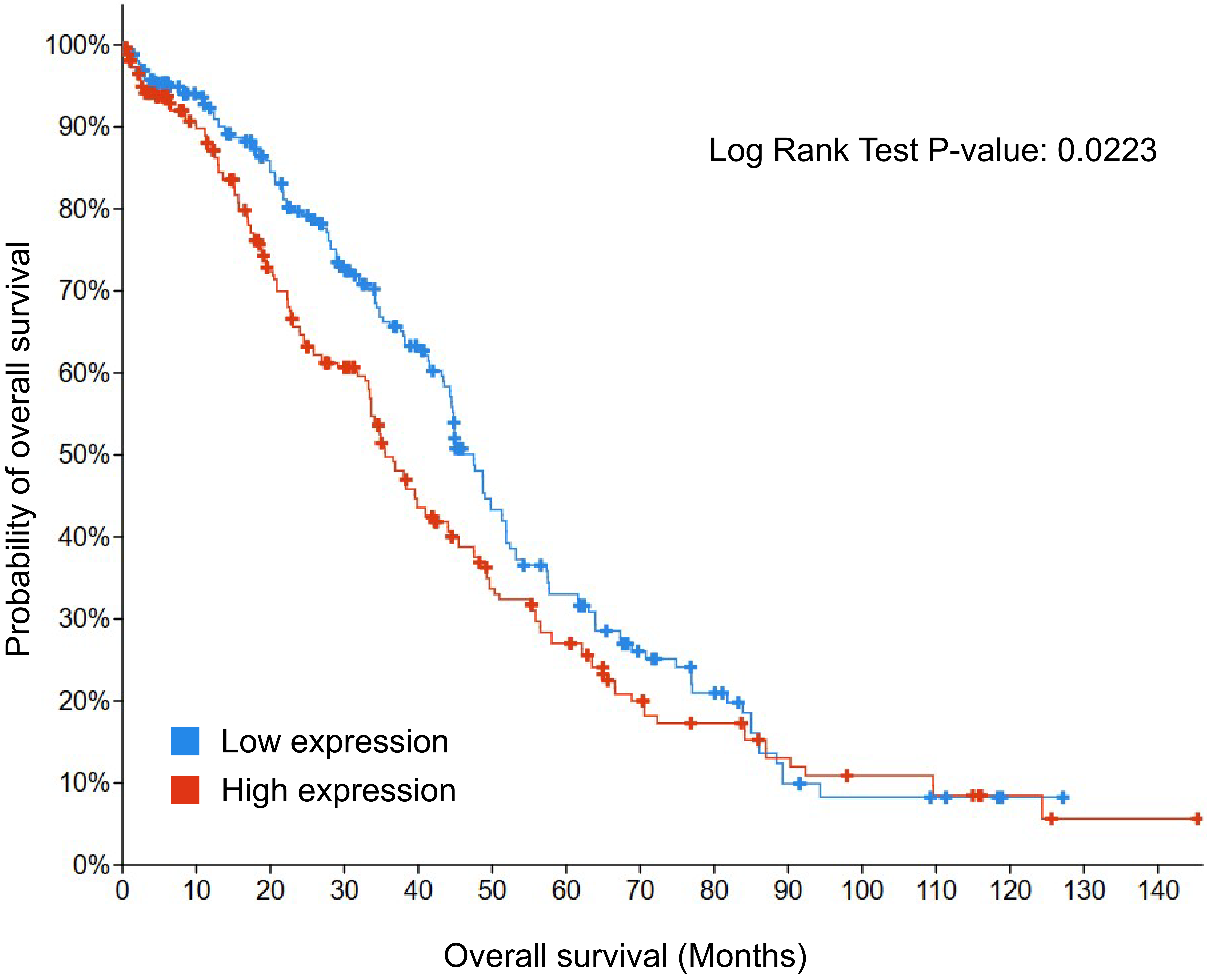

	Number of Cases	Number of Events	Median Months Overall (95% CI)
Low expression (-0.87-0.02)	**260**	**146**	**47.50 (44.51 - 51.87)**
High expression (0.02-0.63)	**260**	**165**	**35.55 (33.64 - 41.98)**
